# Moral and Contextual Dimensions of “Inappropriate” Antibiotic Prescribing in Secondary Care: A Three-Country Interview Study

**DOI:** 10.3389/fsoc.2020.00007

**Published:** 2020-02-20

**Authors:** Carolyn Tarrant, Eva M. Krockow, W. M. I. Dilini Nakkawita, Michele Bolscher, Andrew M. Colman, Edmund Chattoe-Brown, Nelun Perera, Shaheen Mehtar, David R. Jenkins

**Affiliations:** ^1^Department of Health Sciences, University of Leicester, Leicester, United Kingdom; ^2^Department of Neuroscience, Psychology and Behaviour, University of Leicester, Leicester, United Kingdom; ^3^Faculty of Medicine, General Sir John Kotelawala Defence University, Colombo, Sri Lanka; ^4^Tygerberg Academic Hospital and Faculty of Health Sciences, Stellenbosch University, Cape Town, South Africa; ^5^School of Media, Communication and Sociology, University of Leicester, Leicester, United Kingdom; ^6^Department of Clinical Microbiology, University Hospitals of Leicester NHS Trust, Leicester, United Kingdom

**Keywords:** antibiotic prescribing, antimicrobial resistance, hospital, qualitative investigation, international

## Abstract

Overuse of broad-spectrum antibiotics in secondary care is a key contributor to the emergence and spread of antimicrobial resistance (AMR); efforts are focused on minimizing antibiotic overuse as a crucial step toward containing the global threat of AMR. The concept of overtreatment has, however, been difficult to define. Efforts to address the overuse of medicine need to be informed by an understanding of how prescribers themselves understand the problem. We report findings from a qualitative interview study of 46 acute care hospital prescribers differing in seniority from three countries: United Kingdom, Sri Lanka and South Africa. Prescribers were asked about their understanding of inappropriate use of antibiotics. Prescriber definitions of inappropriate use included relatively clear-cut and unambiguous cases of antibiotics being used “incorrectly” (e.g., in the case of viral infections). In many cases, however, antibiotic prescribing decisions were seen as involving uncertainty, with prescribers having to make decisions about the threshold for appropriate use. Decisions about thresholds were commonly framed in moral terms. Some prescribers drew on arguments about their duty to protect public health through having a high threshold for prescribing, while others made strong arguments for prioritizing risk avoidance for the patients in front of them, even at a cost of increased resistance. Notions of whether prescribing was inappropriate were also contextually dependent: high levels of antibiotic prescribing could be seen as a rational response when prescribers were working in challenging contexts, and could be justified in relation to financial and social considerations. Inappropriate antibiotic use is framed by prescribers not just in clinical, but also in moral and contextual terms; this has implications for the design and implementation of antibiotic stewardship interventions aiming to reduce inappropriate use of antibiotics globally.

## Introduction

Antimicrobial resistance (AMR) is a health threat with potentially devastating global consequences (O'Neill, 2016). A key contributor to resistance is the overuse of antibiotics in healthcare; there are a range of drivers including unregulated access to antibiotics in the community in some lower-income settings, and unnecessary and excessive prescribing in community and hospital settings. Previous research indicates that more than one third of antibiotic prescriptions for hospital patients globally may be inappropriate (Zarb et al., 2010).

Appropriate prescribing choices are typically defined as the right drug, administered at the right time, using the right dose, for the right duration (Dryden et al., 2011). Antimicrobial stewardship interventions in hospitals focus on reducing the excessive use of antibiotics, and avoiding the use of inappropriate types of antibiotic, broad-spectrum antibiotics in particular (Hood et al., 2019). Broad-spectrum antibiotics are effective against a wider range of pathogens than narrow-spectrum antibiotics. While they are typically necessary in situations where information is lacking about the cause of an infection, broad-spectrum antibiotics come at the cost of being stronger drivers of AMR (Karam et al., 2016), and ideally their use should be limited to emergency cases (e.g., severe sepsis of unknown origin). Stewardship programmes have been implemented in hospitals worldwide, although with more difficulty in some contexts (Cox et al., 2017; Charani et al., 2019), resulting in positive but variable impact (Hulscher and Prins, 2017; Nathwani et al., 2019).

One challenge for stewardship is that it may be difficult to pinpoint what inappropriate or excessive antibiotic use means in practice, although efforts have been undertaken to try to develop consensus definitions and quality indicators for antibiotic prescribing (Spivak et al., 2016). Defining and measuring inappropriate or suboptimal use is complicated by the tensions that exist between the aim of reducing antibiotic prescribing in order to tackle the growing systemic problem of AMR, and the risks of failing to administer medication to individual patients when there is a potential risk of mortality and morbidity (Fitzpatrick et al., 2019). While prescribing antibiotics in the absence of bacterial infection is clearly inappropriate, clinicians have to base the majority of initial prescribing decisions on clinical judgement–prescribing empirically based on indicative signs and symptoms as opposed to a definitive diagnosis. This is particularly the case for acute medical patients presenting with a spectrum of symptoms that could possibly be indicative of infection. This initial decision could be supported by guidelines and anti-biograms (Liang et al., 2016) where available, and subsequently be refined based on microbiological results or review as part of a hospital's stewardship programme. For individual physicians, however, making these initial treatment decisions under conditions of uncertainty often involves balancing risks; their views about what constitutes the “correct” or most appropriate course of action may differ.

One of the underlying challenges to antimicrobial stewardship is a lack of agreement amongst physicians on what constitutes a “right” choice when making decisions about whether or not to prescribe an antibiotic, and whether to use a broad-spectrum antibiotic as the primary treatment. This variety of conflicting opinions may be grounded in different contextual influences of their medical training, past clinical experiences and current work situation, as well as their orientation toward the uncertainties and risks involved in managing patients with potentially serious conditions.

Drawing on qualitative interview data involving prescribers from a range of different international hospital contexts, this article aims to provide insights into the opinions held by prescribers about what counts as inappropriate prescribing, and the factors that mediate their judgements.

## Methods

### Design

This study used a qualitative interview design, involving interviews with prescribers in secondary care in Sri Lanka, South Africa and the United Kingdom. Semi-structured interviews were conducted between 2016 and 2017. Interviews were conducted in each country by local researchers. We used a detailed shared topic guide (see Appendix A), containing 17 questions about antibiotic use. The guide included questions exploring a range of aspects of antibiotic use, with several questions focusing specifically on identifying the participant's understanding of inappropriate prescribing and asking for examples. In developing the guide we drew on previous research into the determinants of prescribing in hospitals (Krockow et al., 2019), as well as theoretical literature on social dilemmas as this was our overarching theoretical perspective for the study (Tarrant et al., 2019). We piloted and revised the topic guide based on interviews with two junior doctors. We conducted in-depth training and practice interviews for researchers, and held regular telephone meetings to discuss emerging findings through the course of data collection. The interviews were audio recorded and ranged in length between 20 and 80 min. Written consent was obtained from participants for recording of interviews and use of anonymised quotes in reports and publications. All data were anonymised prior to analysis, and participating institutions were offered debriefs about the research findings. Ethical approval was obtained separately in Sri Lanka, South Africa and the United Kingdom.

### Participants

Our study participants included prescribers from three different countries (Sri Lanka, South Africa and the United Kingdom), recruited from a total of seven different hospitals across the three countries. These countries and participating hospitals were selected based on existing collaborations between the research team, and included high and lower resource settings, with diverse challenges in terms of resourcing and patient population.

In Sri Lanka and South Africa a significant proportion of medical care happens in the private sector (in Sri Lanka around 50% of outpatient and 10% of inpatient care is in the private sector (The Economist, 2014), and doctors commonly work across both sectors; around 20% of the South African population are seen in the private sector; Meyer et al., 2017) In both these countries we included public and private hospital settings to explore how these different contexts shaped prescribing. Public health care makes up the majority of care the United Kingdom (Klein, 2005) (and is often used combination with public healthcare) hence both hospitals chosen for the study were public (National Health Service) hospitals. In Sri Lanka we included one private hospital and one public hospital located in a major city, and a publicly funded hospital located in a rural area. In South Africa we included two different hospitals located in a major city. One hospital was publicly funded while the other belonged to a chain of private hospitals. The two hospitals in the UK included a large city teaching hospital and a smaller hospital in an urban area.

Recruitment of participants was conducted using a snowball sampling approach: researchers were introduced to potential participants via email or personal introduction by the local contact in each hospital, or by previous interviewees. We aimed to purposively sample participants to include prescribers with different roles and levels of seniority. We aimed for a minimum sample size of 12 participants per country (total of 36 participants) as our previous experience indicted that this would be a reasonable number to enable us to fully explore the issues. We continued to recruit participants to interviews in each country until the team agreed we had reached a point of data saturation (Aldiabat and Le Navenec, 2018).

### Data Analysis

All interview recordings were transcribed verbatim and anonymised data were analyzed by the United Kingdom-based research team using the constant comparative method (Charmaz, 2014). Starting with open, descriptive coding of a selection of transcripts, an initial coding framework was created using NVivo Software. This was followed by an iterative process of coding and evolution of the coding framework, with reference to existing literature and theoretical concepts (Tarrant et al., 2019). Drawing on this coded data, we focused on codes specifically pertaining to participants' understanding of inappropriate antibiotic prescribing. We generated data summaries for key themes. Visual methods were used to display data extracts and clusters of codes, and to map themes.

## Results

### Participants

We interviewed a total of 46 participants: 18 participants in Sri Lanka, 13 participants in South Africa, and 15 participants in the United Kingdom. The majority of participants were doctors and ranged in seniority from junior doctors to consultants. In the United Kingdom, two advanced nurse prescribers were also included in the sample.

### Findings: Definitions of Inappropriate Antibiotic Use

Our findings highlighted diverse definitions of inappropriate use. There was consensus that some cases of antibiotic use could be seen to be objectively “incorrect” based on the patient's condition or symptoms, but participants' accounts demonstrated that there was often significant ambiguity and lack of consensus about what they judged to be a “correct” decision. Excessive antibiotic use, and high levels of reliance on broad spectrum antibiotics could be justified based on arguments about the duties of a doctor/healthcare professional to their individual patients, and as being appropriate given the local context.

#### “Incorrect” Use of Antibiotics

Prescriber definitions of inappropriate use included examples of relatively clear-cut and unambiguous cases of antibiotics being used “incorrectly.” These definitions included situations where antibiotics were prescribed but where infection was unlikely to be the cause of symptoms, for example, in cases in which symptoms or patient presentation indicated a different root cause such as a viral infection. Indeed, the vast majority of study participants across all countries and hospitals discussed detailed examples of patients being treated with antibiotics for viral illnesses such as the flu. This was seen as problem for patients in primary care settings, but also in hospitals, particularly in ambulatory emergency care.

I think the most common scenario, too common personally in my experience, where […] antibiotics in general are prescribed inappropriately, are viral illnesses. […] especially in, in the ambulatory [emergency] care setting. (UK 001) Some patients clearly having viral infections but they are on antibiotics. (SL 013)

A related type of inappropriate prescribing described by participants was the use of antibiotics in the absence of any symptoms pointing to a bacterial infection. For example, participants reported cases where the mere acuity of a patient triggered a prescription of antibiotics despite the absence of any infection-specific symptoms.

They just come into the emergency unit, and [they are…] getting antibiotics, even though they have a multitude of other reasons for their admission. (SA 012)

So the inappropriate use will be you don't have any evidence that the patient's having bacterial infection. The patient might be unwell due to other reasons, for example they might have asthma exacerbation with very little evidence of infection. (UK 007)

Participants also pointed to situations in which the diagnosis was unambiguous, where clear guidelines about antibiotic choice, dose, and duration existed, but the prescriber failed to prescribe in accordance with these guidelines without justification–resulting in the patient receiving an inappropriate antibiotic or the incorrect dose or duration of treatment.

If there's a clear clinical scenario of infection that we know this is hospital-acquired pneumonia, and you know what kind of antibiotic is that, and you start prescribing a very broad spectrum, then you are not following guidelines, then you are just harming the patient. (SA 009)

Overall, participants from all hospitals and countries shared similar opinions about what constituted an unambiguously clinically “incorrect” decision about antibiotic prescribing, or suboptimal antibiotic use. These types of incorrect or suboptimal uses of antibiotics were commonly seen as reflecting unjustified individual preferences and habits, a lack of appropriate knowledge, or, for more junior doctors a lack of experience or senior supervision. Organizational systems and processes were also seen as playing into this, for example, a lack of access to guidelines, workload and demand on practitioners, or inefficient systems for monitoring and regulating antibiotic use. Participants shared the view that these types of incorrect uses could and should be tackled to reduce antibiotic overuse.

#### Ambiguities of Inappropriate Use: Uncertainty and Moral Framing of Antibiotic Prescribing Decisions

Beyond these shared definitions of incorrect antibiotic use, participants described gray areas of inappropriate use, where the appropriateness of prescribing decisions was less objectively clear. Participants recognized that many antibiotic prescribing decisions involved decision-making under uncertainty, where clinicians were using their clinical judgement to assess the likelihood of infection, the likely source of infection and infective agent, and therefore the best course of action. Prescribers had to make decisions about the threshold at which they would prescribe antibiotic treatment, and their certainty over whether they could use a targeted narrow-spectrum antibiotic as opposed to a broad spectrum antibiotic. This threshold might vary from patient to patient, depending on their vulnerability and level of risk (e.g., young children, frail older people), but also individual prescribers were seen to vary in their approach.

[There] are generally two camps that you get with dealing with uncertainty. So you get the one which is very prone to jump in and do something, and that might be prescribing antibiotics […], which may or may not be appropriate. And then you get the other, which is more likely to just, to try to investigate and work out what's happening before giving an antibiotic. (UK 004)

Under conditions of uncertainty, where judgements had to be made about where to set a threshold for prescribing, antibiotic use was less easy to classify as appropriate or inappropriate in objective terms. Participants recognized that setting a low threshold–i.e., erring on the side of caution and prescribing antibiotics to acutely ill patients “when in doubt,” was an easy and low risk approach to avoiding the risks of deterioration and death for their patients. They also recognized, however, that overuse of antibiotics had negative consequences for society by contributing to the problem of AMR. The tension between the interests of different stakeholders was well-understood by most. Where prescribers were prepared to set the threshold was seen as reflecting, to some extent, their experience and confidence in assessing risk and tolerating uncertainty.

When I started working in this setting, I would be very over-careful of missing something. I think, as I got more confident, I start the conversation with “I don't like prescribing antibiotics. If I feel your child needs an antibiotic, I will give it. But I would prefer to rather wait and see” (SA 007)

In considering how they judged whether their own, and others', levels of prescribing were inappropriately high or low, participants on drew on moral arguments in relation to balancing the interests of the different stakeholders. These arguments reflected participants' underpinning beliefs about what it meant, for them, to be a good doctor or good healthcare professional, and resulted in nuanced, and sometimes contradictory, accounts, of what was inappropriate and why.

Some participants made moral arguments about the importance of considering their duty to broader society (and protecting public health) when making decisions about antibiotic use under uncertainty. For some, appropriate antibiotic use was seen as being grounded in a consideration of the risks to society of excessive antibiotic use, balanced against their duty to their individual patients.

It's kind of a public health like obligation, isn't it, to make sure that you're giving decent antibiotics correctly, to reduce resistant strains. (UK 009)

So we have to balance that risk constantly. And I would say obviously people can argue that your individual patient takes priority, but then other considerations would be society as a whole, or the broader community has to be taken as the priority. […] You have to be cognizant of the fact that these treatment decisions you make on this patient has an impact on the next one and society as a whole. (SA 010)

They made critical judgements about other clinicians who were quick to prescribe antibiotics or relied too heavily on broad spectrum antibiotics.

For the vast majority of cases, the use of broad-spectrum antibiotics is […] a consequence of lackadaisical or poorly worked up clinical decision-making. […] You've got a better chance of getting [the patient] better quicker, because you're covering all possible ills. But it's not good medicine. (UK 002)

While this reasoning sits in line with broader goals of antibiotic stewardship, conversely, other participants made counterarguments to this position, also grounded in moral terms. Although recognizing the tensions in balancing the interests of individuals and society, some participants framed their duty, and correspondingly their understanding, of what it meant to be a good doctor or healthcare professional, in terms of prioritizing the wellbeing of the individual patients in front of them. Although they recognized the clinical importance of treating patients appropriately, they minimized the risk of AMR and their responsibility for the problem, in comparison with the risks and their responsibilities for sick patients in front of them.

I think I try to do what is good for the patient and that is the only thing […] The only agenda I have is that. (SL 007)

As a doctor, the most important is the patient's interests, you know, so you try and do the right thing for that specific patient, and then, the other interests are probably less important. (SA 006)

I have that sense at a societal level [of the problem of AMR], but my job as a doctor is to treat the person in front of me. […] so I don't balance… […I'm] just doing what I can to make the patient better. (UK 012)

These participants drew on such arguments as justification for using antibiotics, and particularly broad spectrum antibiotics, when in doubt, even if this was at a cost of increased resistance.

As a doctor we need to save patients, […] even if we think that this is broad-spectrum antibiotics, and [we should be concerned about] resistance with the [hospital] trust, but at that time I think the most important thing is to treat your patient well. (UK 005)

As such, judgements about the appropriateness of antibiotic prescribing decisions could not always be pinned down in objective clinical terms. Instead, how participants justified their approach to antibiotic prescribing under conditions of uncertainty reflected their orientation to risk, and their position about what it meant to be a good doctor in terms of moral responsibilities. Participants took different standpoints in relation to where their duties as a doctor or healthcare professional lay, and therefore, what was and was not appropriate practice.

#### Ambiguities of Inappropriate Use: Inappropriate Prescribing as Contextually Dependent

Notions of whether levels of antibiotic prescribing were considered to be inappropriate were also contextually dependent: what could potentially be seen as over-use of antibiotics, or excessive reliance on broad-spectrum antibiotics, was re-framed in some cases by participants as a rational and appropriate response to the conditions in which they worked. Although they recognized such antibiotic use as excessive, they did not always see it as inappropriate in the context of the demands they faced and the resources available to them. This was a particularly common response from participants in low resource settings.

Such challenging circumstances in low resource settings included conditions of high patient throughput–including high numbers of patients presenting at a late stage when they were acutely ill. Problems also arose when patients who had already taken (often unspecified) antibiotics in the community prior to coming into hospital–including antibiotics that had been prescribed without any microbiological testing, or had been purchased. This constrained the choices about how these patients could be treated once they arrived in hospital. It also meant that that waiting for microbiological tests prior to prescribing was commonly seen as futile, and this futility was exacerbated in some contexts by the lack of rapid and high quality testing services.

When the patients come very late [i.e., present at hospital with infections at an advanced stage] by that time they will have at least more than one system affected. […] so we will again be using the broad spectrum even without [waiting] for the cultures and things like that (SL 003)

*Unfortunately, because our diagnostic tests are not that great, and turnaround times are poor, and sensitivities etc. are not that good, you might have to go [with] broad [spectrum antibiotics] (SA 012*)

Unsanitary and overcrowded environments were seen as vastly increasing the risk of hospital-associated infections, increasing the need to rely on antibiotics.

We are seeing a lot of […] infections in our wards because we [don't] have the facilities, I mean like the beds are very close and they are not in separate parts, cubicles. (SL 015)

In these cases, it was not the antibiotic use that was seen as wrong or inappropriate, but the precipitating conditions. Participants felt they were able to respond to these conditions only in the best way that they could. While these problems–access to hospitals and use of antibiotics in the community, and insanitary hospital environments–remained as they were, participants were able to justify high levels of antibiotic use and saw little opportunity for reducing their use.

Other contextual factors that shaped views about whether antibiotic prescribing was the “right” thing to do, even at a cost of increased resistance, related to financial and social considerations. Although some participants flagged the problem of costs of excessive use of antibiotics for their organizations, others argued that financial and social considerations for patients could make antibiotic use the appropriate choice. In private hospitals, some argued that using broad-spectrum antibiotics could help reduce costs for patients arising from length of stay. In lower income settings, participants recognized that a hospital stay could be financially devastating for wage earners in families and had an impact on the economy: treating patients aggressively to get them well and out of hospital quickly was seen in some cases as a priority.

Most people with private medical insurance, they have to […] get out of the hospital as soon as possible. They work for themselves, a lot of the people, they cannot afford to stay long in the hospital. So if you give […] a good broad-spectrum antibiotic to start off with […] it's a win-win situation all the way. (SA 004)

Broad-spectrum benefit was, […] start medications, there will be improvement, so these people are working, I mean, going back to working. (SL 005)

## Discussion and Conclusions

The interview findings from doctors and nurse prescribers across three countries and different hospital sites suggest ambiguities in opinions about what counts as inappropriate antibiotic prescribing and antibiotic over-use in hospital settings. Our focus in this paper was on how prescribers made judgements about the appropriateness of antibiotic use, and how they justified their own and others' use of antibiotics. In terms of prescribers' own understanding of appropriateness, there was consensus that antibiotic use under certain circumstances could be judged to be clinically “incorrect” (e.g., “incorrect” the use of antibiotics for viral illnesses). Not all decisions about antibiotic use could, however be judged as objectively appropriate/inappropriate in clinical terms. There was significant ambiguity about judgements of appropriateness of antibiotic use in case of diagnostic uncertainty. Such judgements were mediated by personal perceptions of working within the frames of risk and uncertainty, and participants' comfort in tolerating risk. As identified in previous research, this could vary dependent on individual training, experience, and seniority, but also on the extent of concern about the impact of negative patient outcomes, and the risk of personal and reputational damage (Krockow et al., 2019).

Judgements about the appropriateness of antibiotic use also drew on moral reasoning about what it meant to be a good doctor or healthcare professional. This reflects what has been referred to as *relational ethical reasoning*: reasoning aimed at working out “what is the right thing to do” based on an individual's role, and relationship with and responsibility for others (Austin et al., 2003; Pollard, 2015). In the context of medicine, relational ethical reasoning is directed at answering such questions as: What makes a good doctor or health professional? Am I a good doctor or healthcare professional (Lindseth, 1992)? This reasoning reflects not only individual skills and experience, but also how an individual sees themselves as positioned, and where their responsibilities lie, in relation to their patients and other stakeholders (Sørlie et al., 2001). Prescribers see themselves as “acting wisely in the face of inevitable uncertainty” (Tanenbaum, 1993), but make different interpretations of what it means to do so. Individual participants varied in their views of their responsibilities in relation to public health and for considering wider society in their decision making; some felt the individual patient was their only concern. This finding builds on other research demonstrating that individual prescriber decisions about antibiotic use are underpinned by different perceptions: the extent to which they are oriented toward AMR and infectious diseases (Björkman et al., 2010) as opposed to having a dominating focus on the care of the patient. This tension between attending to the needs of individual patients vs. tending to the needs of the population as a whole has been recognized as a central ethical problem in diverse areas of medicine, particularly preventative medicine (Rosenberg, 1998; Griffiths et al., 2006). Our study highlights how this tension underpinned moral judgements about antibiotic use: what one prescriber judged to be excessive antibiotic use, based on their perceptions of duty to consider public health, could be seen by another as an appropriate response based on their sense of responsibility to minimize risk to the individual patient in front of them. These findings raise questions about what good practice can mean within existing health care systems: with attendant regulatory and structural drivers that prioritize immediate patient outcomes; and formalized ethical principles for professional practice (General Medical Council, 2019; Sri Lanka Medical Association, 2019)^1^ that define being a “good doctor” in terms of making the care of the individual patient their primary concern, and protecting the life of their patients.

Judgements about the appropriateness also reflected the context within which prescribers were working: high levels of antibiotic use could be seen as a rational and morally justifiable response to challenging conditions such as patient acuity and poor environments in hospitals for hygiene and infection control. The importance of cultural and contextual factors in shaping antibiotic use is well-recognized (Hulscher et al., 2010,?; Pearson et al., 2018; Wilkinson et al., 2019): our study shows how these factors also played into prescribers' reasoning about appropriateness of antibiotic use. “Excessive” antibiotic use could be recognized as such by prescribers but nonetheless be seen as representing a reasonable response to local conditions. In this sense, although levels of prescribing were seen as excessive, they were not seen as inappropriate. As such, judgements about the appropriateness of antibiotic use did not solely reflect a fixed individual moral position, but were situated in context of the local systems and structures of care, and the temporality of the patient's presentation. It is apparent that the way doctors and other prescribers make judgements about appropriateness are grounded in individual moral reasoning, and are highly contextualized: they cannot be reduced to purely technical criteria.

Our study has limitations. We included participants from three countries, including high and lower resource settings, hence the generalizability of our findings to other international settings is necessarily limited. We conducted interviews with a small number of participants in each hospital, although we included prescribers with different roles and different levels of seniority and experience. Our study design did not allow us to explore how practitioners actually behaved in practice in relation to decision-making about antibiotic use. Also, our analysis focused specifically on antibiotic prescribing decisions; we did not explore other dimensions of antibiotic use such as medication review, stopping or switching antibiotics. Reviewing antibiotic prescribing is an important focus for stewardship, providing a way of updating or correcting initial prescribing decisions particularly in the light of new information that can provide more certainty about the best clinical course of action. Activities around reviewing, stopping and switching antibiotics present a range of different challenges (Schouten et al., 2007) which were not the focus of our study.

A strength of our study is the inclusion of a range of different organizations across three international contexts, including high and lower-income settings, and public and private hospitals. We did not include a private hospital in the United Kingdom, because the majority of acute healthcare provision is through publicly-funded NHS providers. A further strength is the conduct of the interviews in each locality by local researchers, who were familiar with local health systems and could build rapport effectively with participants. Although contextual factors, patient characteristics, and stewardship activities varied significantly between countries and hospitals, it is notable that we found strong concordance across the settings in terms of definitions of “incorrect” use, and of uncertainty and moral aspects of decision-making. Findings relating to contextual influences mainly came from the interviews in lower-income settings, although NHS staff in the United Kingdom did reflect on some of these considerations including cost to the healthcare system.

Our findings have implications for antimicrobial stewardship. As highlighted earlier in this paper, lack of consensus among prescribers about what constitutes inappropriate use presents a challenge for stewardship efforts. Our findings suggest that this lack of consensus is unlikely to have a technical solution–for example, through drawing up more specific definitions–because judgements about appropriateness are morally and contextually framed. Stewardship interventions that directly target behavior change using techniques such as education, restrictions and controls on prescribing, and audit and feedback (Davey et al., 2017) may have value where there is consensus that prescribing is wrong or suboptimal. These types of interventions may, however, be less effective at addressing the underpinnings of moral reasoning about antibiotic use, or the structural and contextual factors, that from the point of view of prescribers can make antibiotic overuse a rational and justifiable action. Aiming to tackle inappropriate prescribing may be problematic where consensus is lacking about what in fact constitutes “inappropriate” prescribing: where this phenomenon is morally contestable and contextually-embedded. The terminology of “inappropriate” or “suboptimal” prescribing itself may be unhelpful, given the implicit assumption that this can always be judged objectively based on the facts of the matter.

One implication of our findings is that, rather than assuming that inappropriate prescribing can be objectively specified and therefore reduced through simple interventions, there may be a need to look at how to provide more support for prescribers in managing uncertainty. Stewardship approaches that aim to support empirical decision making, improve documentation of rationale for antibiotic use, and focus on reviews of antibiotic prescriptions (based on updated information providing more certainty, such as microbiology results) are clearly important. There is also a need, however, to address the moral aspects of prescribing decisions. This might involve including vignette-based debates in stewardship training, and providing opportunities for collective input to difficult decisions. We may also need more explicit societal debate, and the establishment of collective agreements around, the duty of prescribers to consider the interests of society in making antibiotic prescribing decisions (Tarrant et al., 2019). Consensus guidelines and decision-support tools have been identified as approaches to managing moral dilemmas in antibiotic prescribing (Leibovici et al., 2012). Another implication is the need to recognize that efforts to reduce inappropriate antibiotic use by targeting prescribing behavior (for example, through education, or auditing) may be futile if they fail to conceptualize antibiotic overuse as a rational response to local cultural and contextual conditions. Even antibiotic use that can be objectively defined as “clinically incorrect” could reflect the accepted practice of using antibiotics as a “quick fix” to complex problems such as poorly integrated health systems (Denyer Willis and Chandler, 2019), particularly in resource limited settings. This points to the need for a more holistic approach (McLeod et al., 2019) that considers the broader drivers of antibiotic use in secondary care settings globally, including issues such as sanitation, community healthcare, and the financial implications for patients of hospitalization.

Our study suggests that inappropriate antibiotic use is framed by prescribers not just in clinical, but also in moral and contextual terms; this has implications for the design and implementation of antibiotic stewardship interventions aiming to reduce inappropriate use of antibiotics globally.

## Data Availability Statement

The datasets generated for this study are available on request to the corresponding author.

## Ethics Statement

The studies involving human participants were reviewed and approved by University of Leicester Research Ethics Committee, Sri Lanka Medical Association Ethics Review Committee, and University of Stellenbosch Health Research Ethics Committee 1. The patients/participants provided their written informed consent to participate in this study.

## Author Contributions

CT, AC, EC-B, DJ, NP, SM, and EK planned and designed the study. EK, CT, WN, and MB conducted interviews. CT and EK conducted the data analysis and drafted the first manuscript version. All authors helped to review and refine the final version.

### Conflict of Interest

The authors declare that the research was conducted in the absence of any commercial or financial relationships that could be construed as a potential conflict of interest.

## Footnotes

^1^Africa HPCoS. Ethical guidelines for good practice in the health care professions [Available from: https://www.hpcsa.co.za/Uploads/Professional_Practice/Ethics_Booklet.pdf].

## Appendix A: Topic Guide for Interviews

### Questions About Their Role

Can you tell me briefly about your job role? What is your involvement in the antibiotic prescribing for acute medical patients?

What education or training have you had specifically on antibiotic prescribing?

### Prescribing Decisions

I'd like you to consider antibiotic prescribing for an acute medical patient with a suspected infection, that is, when it is not confirmed that the patient has an infection, or what the infective organism might be (also known as empirical prescribing).

1. How do you go about making the decision whether or not to prescribe an antibiotic?
  - *Are there any* ‘rules of thumb’ *that you use? What influences this decision?*
2. Can you tell me about how you decide which antibiotic to use, for an acute medical patient with a suspected infection?
  - *Local or national* guidelines *on antibiotic prescribing?*
  - Any limitations/restrictions *on the antibiotics you can use?*
  - *Do you ever get* advice *on your prescribing decisions? Who from & why?*
3. How important do you feel it is to collect microbiology specimens, in making antibiotic prescribing decisions? Why?

I would be interested to hear your thoughts on choosing between a broad vs. a narrow spectrum antibiotic. Broad spectrum antibiotics being an antibiotic with activity against a wide range of pathogens. A narrow spectrum antibiotic is one that is targeted at a specific organism.

4. How easy do you find this decision? What do you see as the uncertainties and how do you deal with them? *What sort of influences are there on your decision?*
5. What would you see as the benefits of prescribing a broad spectrum antibiotic (BSA), as opposed to a narrow spectrum antibiotic?
6. What would you see as the risks of prescribing a BSA, as opposed to a narrow spectrum antibiotic?
7. Do different stakeholders have different interests? [patient / doctor / hospital / society] To what extent do you consider these in your day to day prescribing, and how do you balance these interests?
8. If you prescribe a BSA, how likely is it that the patient would be switched to a narrow spectrum antibiotic at a later point? *Why? What are the barriers to this? What helps make it easier?*
9. How do you know whether you are making good decisions about antibiotic prescribing? Do you get any feedback about your antibiotic prescribing approach?
10. Do you ever feel patients are prescribed BSAs inappropriately? Could you start by saying what you see as inappropriate use? Are there common situations where this happens? Why do you think this happens?
11. What steps could be taken to stimulate appropriate use of BSAs?
  - Main barriers to improving the way BSAs are used in this hospital? e.g.: local culture / lack of lab facilities / organizational policies / external incentives or pressure
  I'd like to focus now on antibiotic resistance, that is, the ability of a bacteria to stop an antibiotic from working against it, meaning that some antibiotic treatments become ineffective, infections persist and can spread to others. This can mean having reduced or no antibiotic treatment options. Do you worry about the problem of antimicrobial resistance in your day to day practice? Why?
12. Do you ever see examples of resistance? How often does this happen in your experience?
13. How much does the problem of antibiotic resistance influence your decision-making about prescribing antibiotics?
14. Do you get information about overall levels of antibiotic resistance in this hospital?
15. Do you think that reducing the use of BSAs in hospitals would make an important difference to addressing the overall AMR problem? Why yes or no?

## Ending

Is there anything else you'd like to add about the use of BSAs, or the problem of AMR? Thanks for participating!
